# Nonceliac gluten sensitivity

**DOI:** 10.1097/MCO.0000000000000925

**Published:** 2023-02-15

**Authors:** Carlo Catassi, Giulia Catassi, Loris Naspi

**Affiliations:** a Department of Odontostomatologic and Specialized Clinical Sciences, Polytechnic University of Marche, Ancona; b Pediatric Gastroenterology and Liver Unit, Department of Maternal and Child Health, Sapienza-University of Rome, Rome, Italy; c Department of Psychology, Humboldt University, Berlin, Germany

**Keywords:** dietary treatment, gluten, irritable bowel syndrome, nonceliac wheat sensitivity

## Abstract

To describe recent advances on nonceliac gluten sensitivity (NCGS), a recently described disorder characterized by variable symptoms and frequent irritable bowel syndrome (IBS)-like manifestations.

**Recent findings:**

The recent description of disease-triggering wheat components other than gluten, such as fructans and amylase-trypsin inhibitors (ATIs), definitely suggests that nonceliac wheat sensitivity (NCWS) is a better ‘umbrella‘ terminology than NCGS. Self-reported NCWS is very common worldwide, particularly in patients seen at the gastroenterology clinic, but many of these diagnoses are not confirmed by standard clinical criteria. A biomarker of NCWS is still lacking, however, subtle histological features at the small intestinal biopsy may facilitate diagnosis. Treatment of NCWS is based on the gluten-free diet (GFD). The GFD has proven to be an effective treatment of a significant proportion of NCWS-related IBS patients. Dietary therapies for IBS, including the GFD, should be offered by dietitians who first assess dietary triggers and then tailor the intervention according to patient choice. Pioneer studies are under way to test the therapeutic efficacy of supplemental gluten-digesting enzyme preparations in patients with NCWS.

**Summary:**

Recent studies highlight interesting pathophysiological and clinical features of NCWS. Many questions remain, however, unanswered, such as the epidemiology, a biomarker(s), and the natural history of this clinical entity.

## INTRODUCTION

Nonceliac gluten sensitivity (NCGS) is a poorly defined syndrome characterized by intestinal and extraintestinal symptoms related to the ingestion of gluten in subjects that are not affected by either celiac disease (CeD) or wheat allergy. Diagnosis of NCGS is based on: exclusion of CeD and wheat allergy, (b) demonstration of patient's clinical response to the gluten-free diet (GFD), and (c) confirmation by a gluten/wheat challenge, possibly performed with a double-blind, placebo-controlled procedure [1,2]. As wheat components other than gluten, such as Fermentable Oligosaccharides, Disaccharides, Monosaccharides and Polyols (FODMAPs) and amylase-trypsin inhibitors (ATIs) may elicit symptoms indistinguishable from NCGS, the definition ‘nonceliac wheat sensitivity’ (NCWS), instead than NCGS, is more appropriate according to many experts [3]. Several aspects of NCWS are still unclear, and currently under scrutiny. In this article, we will present a concise review of advances in NCWS over the preceding year, highlighting the most important and interesting articles divided by topic, among the many published within the past 18 months. 

**Box 1:**
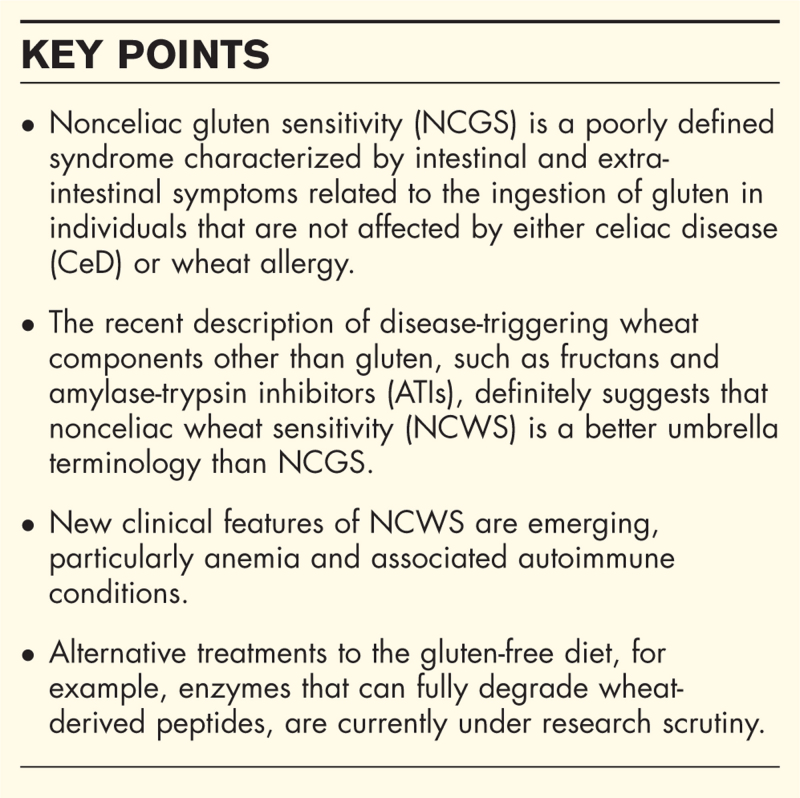
no caption available

## EPIDEMIOLOGY OF SELF-REPORTED NONCELIAC WHEAT SENSITIVITY

Due to lack of a biomarker, the prevalence of NCWS is difficult to establish and ranges between 0.6 and 13% of the general population. Many individuals experience symptoms related to the ingestion of wheat derivatives, and put themselves on a gluten-free diet (GFD) without consulting a doctor. This condition of self-reported NCWS is very common worldwide and particularly frequent in patients seen at the gastroenterology clinic, as confirmed by recent surveys. Among outpatients referred for digestive endoscopy in Italy, 20% reported self-diagnosed NCWS, more frequently female individuals and younger patients [4]. An Indian study showed that 11.3% of 204 patients with irritable bowel syndrome (IBS) and none of 400 healthy controls self-reported wheat sensitivity, and all IBS patients reported clinical improvement after 6 weeks of GFD [5]. Likewise, self-reported NCWS was found in 33.6% of Korean patients with IBS, most frequently complying of bloating, abdominal discomfort, and belching [6]. The relevance of self-reported NCWS remains, however, unclear, as only a minority of these self-diagnosed individuals are affected by true NCWS, as confirmed by appropriate clinical criteria.

## WHAT IS THE TRIGGER OF NONCELIAC WHEAT SENSITIVITY?

The triggering effect of a single dietary component like gluten is difficult to prove in controlled studies, because of an unavoidable, strong nocebo effect. This has been confirmed by a recent blinded, randomized cross-over trial, performed on adolescents and young adults affected with multiple gastrointestinal symptoms that responded to the GFD: in these subjects, the ingestion of 10 g of purified gluten for 1 week, as compared with placebo, did not elicit gastrointestinal symptoms or worsened the outcomes measuring mental health [7^▪^]. On the other hand, it is becoming increasingly clear that wheat components different from gluten may cause symptoms of NCWS. Due to the high content of fructans (0.7–2.9 g% in wheat flour), wheat is one of the major sources of dietary FODMAPs, a group of poorly absorbed carbohydrates that increase small bowel water content by osmosis and release gases, predominantly carbon dioxide and hydrogen, from bacterial fermentation. Over the last decade, there has been considerable interest in the role of the low FODMAP diet (LFD) to manage patients with IBS, a functional intestinal disorder largely overlapping with NCWS [8]. A recent systematic review on the effectiveness of the LFD in improving NCWS concluded that the GFD represent first-line therapy but also that a complete FODMAP restriction (not only wheat fructans) can further decrease gastrointestinal symptoms in individuals with NCWS [9]. Amylase/trypsin inhibitors (ATIs) constitute about 2–4% of the total wheat grain proteins and have been implicated in adverse reactions to wheat exposure [10^▪^]. ATIs are innate triggers of intestinal and extra-intestinal immune activation after ingestion of gluten-containing foods. ATIs pass the intestinal epithelium as intact proteins and stimulate the toll-like receptor 4 (TLR4) on the lamina propria monocytes, macrophages (MΦ), and dendritic cells. Despite the interesting results of some in-vitro and animal studies, so far, no controlled human interventions have been carried out with well characterized purified ATI fractions isolated from processed wheat-containing foods. Consequently, the potential role of ATIs in the pathophysiology of NCWS remains unclear [11]. In conclusion, it appears that several wheat components (currently including gluten, FODMAPs and ATIs), acting alone or in synergy, may activate different mechanisms involving the innate and adaptive immune response and/or a metabolic pathway, finally leading to the clinical picture of NCWS or other wheat-related disorders (CeD and wheat allergy). As the GFD implicates the elimination or a drastic reduction of all these wheat components, it may be difficult to identify the ‘true’ culprit/s at the patient’ level.

## EMERGING CLINICAL FEATURES OF NONCELIAC WHEAT SENSITIVITY

Typical clinical features of NCWS include IBS-like intestinal symptoms, particularly bloating, abdominal pain and diarrhea, and neurological complaints, such as headache, ‘foggy mind’ and chronic fatigue [1]. However, many different presentations of NCWS have been described [2], and new clinical features are still emerging.

By analysing data from 244 NCWS patients diagnosed by double-blind placebo-controlled wheat challenge, it has recently been shown that anemia prevalence is significantly more common (34.8%; mean hemoglobin 10.4 ± 1.4 g/dl), than in IBS (17.4%). Microcytic/hypochromic anemia and altered iron metabolism in NCWS can be treated with a long-term strict wheat-free diet. Authors suggest that NCWS should be included in the differential diagnosis of anemic patients with ’functional gastrointestinal troubles‘ [12].

Mansueto and co-workers recently investigated the frequency of autoimmune diseases and autoantibodies in 91 patients (78 women) with NCWS. The frequency of autoimmune diseases was higher in patients with NCWS than in healthy controls and IBS patients. In the NCWS group, antinuclear antibodies tested positive in 71.4% vs. healthy controls (19.7%) and IBS patients (21.8%). The frequency of extractable nuclear antigen antibody (ENA) positivity was significantly higher in patients with NCWS (21.9%) than in healthy controls (0%) and patients with IBS (3.6%). Among patients with NCWS, 9.9% tested positive for antithyroglobulin, 16.5% for antithyroid peroxidase, and 14.3% for antiparietal cell antibodies. In summary, one in four patients with NCWS suffered from autoimmune diseases, and serum antinuclear antibodies were positive in a very high percentage of cases. These data show that NCWS may be associated to autoimmune diseases, even though the mechanism of this comorbidity remains unclear [13^▪^].

## NEW BIOMARKERS OF NONCELIAC GLUTEN SENSITIVITY/NONCELIAC WHEAT SENSITIVITY

The diagnosis of NCWS is heavily dependent on the clear-cut demonstration of causality between wheat ingestion and consequent appearance of symptoms, as originally standardized by the so-called Salerno diagnostic criteria [2]. However, several gluten/wheat re-challenge studies have shown that these clinical criteria are poorly specific, particularly because of a strong nocebo effect of the food challenge and the possible confounding related to different triggers of NCWS [3]. For this reason, the search of a biomarker of NCWS remains very active.

Zonulin (haptoglobin 2 precursor) is a protein that increases the permeability of tight junctions between enterocytes. Dysregulation of the zonulin pathway and subsequent ‘gut leakiness’ because of increased intestinal permeability has been associated with the pathogenesis of gastrointestinal disorders, such as CeD, NCWS/IBS, and inflammatory bowel disease [14]. In a recent, multicentre Italian study, the diagnostic accuracy of serum zonulin determination was evaluated in 86 patients with either self-reported or double-blind confirmed NCGS, 59 patients with diarrhoea-predominant IBS (IBS-D), 15 patients with CeD and 25 asymptomatic controls. The diagnostic accuracy of zonulin levels in distinguishing NCGS from IBS-D was 81%. After exclusion of CeD, a NCWS diagnostic algorithm combining zonulin levels, symptoms and gender improved the accuracy to 89% [15^▪^]. These results are, however, at variance with a previous study showing that serum zonulin as a biomarker fails to identify the IBS, functional dyspepsia and NCWS [16]. Furthermore, doubts have been expressed about the reliability of commercial ELISAs used to measure zonulin in blood and stool [17]. Therefore, the utility of zonulin measurement in the clinical setting remains to be established.

A recent work evaluated the diagnostic accuracy of confocal laser endomicroscopy (CLE) for the identification of NCWS. CLE generates high-resolution images of the gastrointestinal tract after intravenous injection of fluorescein during ongoing endoscopy. This technique demonstrated clinical impact in a variety of gastrointestinal diseases. During the baseline CLE and after food provocation testing, authors used the fluorescein leakage and increased intervillous spaces as positive diagnostic criteria. As only 34 of 74 patients with NCWS and 38 of 56 non-NCWS controls were correctly identified by CLE, authors concluded that the diagnostic accuracy of CLE is too low to recommend widespread use of this invasive procedure [18].

From the histological perspective, NCWS has been classified as gluten sensitivity with normal or nearly normal aspect of the small intestinal mucosa. However, the histological hallmarks of NCWS, if any, are still poorly defined. New data on NCWS histology have been collected in a worldwide multicenter study addressed to quantify the morphological changes in NCWS as compared with controls and to patients with CeD with milder enteropathies (Marsh I-II), with the aim of delineating the histological spectrum of NCWS and its distinction from normal intestinal mucosa. The median villous height in NCWS was significantly shorter (600 μm) than controls (900 μm). The villus-to-crypt ratio (VCR) in NCWS with Marsh 0 was lower than controls. The median intraepithelial lymphocyte (IEL) count in NCWS with Marsh 0 was higher than controls (23.0 vs. 13.7 per 100 enterocytes). To distinguish Marsh 0 NCWS from controls, an IEL cut-off of 14 showed 79% sensitivity and 55% specificity. IEL densities in Marsh I–II NCWS and CeD groups were similar. Authors conclude that small bowel histology of NCWS exhibits minimal changes suggesting an intestinal response to luminal antigens, even at the Marsh 0 stage of villus architecture. These features are subtle and not disease-specific but might be helpful in the diagnosis of NCWS [19^▪▪^].

## DIETARY TREATMENT OF NONCELIAC WHEAT SENSITIVITY-RELATED IRRITABLE BOWEL SYNDROME

As previously observed, there is a well established overlap between NCWS and IBS in adult patients [1]. In the Spanish study by Fernandez-Bañares *et al.*[20], as many as 62% of IBS patients ameliorated after starting treatment with the GFD. However, this work does not allow any firm conclusion on causal effects of the GFD because of lack of a control group. Dietary therapies are frequently recommended in IBS, with more than 80% of patients reporting food-related symptoms [21^▪▪^]. A recent randomized trial compared the short-term efficacy of traditional dietary advice (TDA), based on reducing alcohol and caffeine intake, avoidance of spicy meals, reduction in fat intake and increase in fluid intake as well as soluble fibre intake, against the LFD and the GFD in patients affected with nonconstipated IBS. The primary end-point of at least 50-point reduction in IBS symptoms severity score (SSS) was met by 42% undertaking TDA, 55% for LFD, and 58% for GFD. Responders had similar improvements in IBS-SSS items regardless of their allocated diet. Individuals found TDA cheaper, less time-consuming to shop, and easier to follow when eating out than the GFD and LFD. TDA was also easier to incorporate into daily life than the LFD. Authors recommend TDA as the first-choice dietary therapy in nonconstipated IBS, with LFD and GFD reserved according to specific patient preferences and specialist dietetic input [21^▪▪^]. As previously noted, there is overlap between the LFD and the GFD, in that the GFD eliminates wheat derivatives that are also the major source of FODMAPs (fructans), and IBS patients often self-initiated gluten/wheat reduction as part of their LFD. To avoid this confusing situation and optimize dietary treatment, a recent UK Consensus document recommends that (a) dietary therapies for IBS should be offered by dietitians (instead than physicians/gastroenterologists) who first assess dietary triggers and then tailor the intervention according to patient choice; (b) novel approaches such as employing group clinics and online webinars may maximize capacity and accessibility for patients [8]. Further research is required to assess the comparative efficacy of dietary therapies to other management strategies available to manage IBS.

## THE ROAD AHEAD: ALTERNATIVE TREATMENT TO THE GLUTEN-FREE DIET

Gluten-containing food, particularly wheat, has a primary role in the diet of many populations worldwide. Therefore, the GFD diet may have a negative impact on the psychosocial aspects of daily life [22^▪^]. For this reason, new ways of treating gluten-related disorders, other than the GFD, are currently under research scrutiny. The selection of wheat varieties containing no/reduced immunogenic gluten proteins could improve the quality of life of people on the GFD. Conventional mutation and breeding methods were not successful, but new techniques of gene silencing (RNAi) or editing (CRISPR/Cas9 = clustered regularly interspaced short palindromic repeats/CRISPR-associated protein 9) represent an interesting new approach [23].

Supplemental enzyme preparations can effectively accelerate the breakdown of protein and hydrolysis of toxic gliadin fractions from the early stages of gastric digestion, thereby reducing intestinal exposure and potentially limiting the adverse reaction to gluten [24]. A recent randomized trial evaluated the efficacy of endopeptidase P1016 in neutralizing the effects of a controlled reintroduction of gluten in NCGS patients. Unfortunately, during gluten reintroduction, patients reported a significant increase in abdominal pain and a worsening of stool consistency, with no difference between patients receiving the endopeptidase or the placebo [25^▪^]. As previously noted, ATIs may be responsible, at least in part, of clinical symptoms of wheat intolerance. Recently, a digestive supplement enriched in caricain, an enzyme naturally present in papaya latex originally designed to act against gluten proteins, was assessed for its ability to digest wheat ATIs. Caricain showed rapid action *in vitro* against known immunogenic ATIs, indicating its potential utility for digestion of wheat ATIs in the upper digestive tract [26].

## CONCLUSION

Within the spectrum of GRD, NCGS/NCWS is a quickly evolving area. This is hardly surprising, given the short period of time since the first clinical and immunological characterization of NCGS appeared in the scientific literature in the year 2010. The recent description of disease-triggering wheat components other than gluten, such as fructans (belonging to FODMAPs) and ATIs, definitely suggests that NCWS is a better ‘umbrella’ terminology than NCGS. From a clinical point of view, the overlap with IBS is a challenging feature of NCWS that is currently the focus of intense clinical research. The possibility to personalize the dietary treatment of IBS can improve the quality of life of a large number of patients affected with this common disorder.

From a broad perspective, there is no doubt that a number of individuals who are not affected with CeD or wheat allergy may experience an improvement in their symptoms and quality of life after starting the gluten/wheat-free diet. However, it is also unquestionable that several aspects of this NCWS, particularly the epidemiology, a biomarker, and the natural history, remain undefined. For this reasons, further high-quality studies are urgently required to address the many questions that still remain unanswered about NCWS.

## Acknowledgements

*None.*

### Financial support and sponsorship

*None.*

### Conflicts of interest

*C.C. has received honoraria from Dr Schaer Food Company and Noos s.r.l. For the remaining authors none were declared.*

## REFERENCES AND RECOMMENDED READING

Papers of particular interest, published within the annual period of review, have been highlighted as:

▪ of special interest
▪▪ of outstanding interest
